# Editorial: New challenges in animal welfare, volume II

**DOI:** 10.3389/fvets.2024.1520099

**Published:** 2024-11-21

**Authors:** Rodrigo Muiño, Elena Niceas Martínez, Joaquin Hernandez, Jose Luis Benedito, Cristina Castillo

**Affiliations:** Department of Animal Pathology, Faculty of Veterinary Medicine, IBADER-University of Santiago de Compostela, Lugo, Spain

**Keywords:** animal welfare, human-animal relationship, environmental enrichment, stress and handling, assessment protocols

Animal welfare has become an increasingly important concern in recent years, as society's awareness and ethical standards regarding the treatment of animals continue to evolve. Today, the field of animal welfare faces a new set of challenges that demand innovative solutions and a renewed commitment to improving the wellbeing of animals across various sectors (1). These emerging issues range from the impacts of climate change and the complexities of modern farming practices to the ethical considerations of animal research and the shifting dynamics of pet ownership in the post-pandemic era (2). To address these challenges, animal welfare organizations, researchers, policymakers, and the public must collaborate to approach the multifaceted aspects of animal welfare holistically (3)

This Research Topic of *Frontiers in Veterinary Science*, titled “*New Challenges in Animal Welfare*,” compiles research that examines diverse approaches for assessing and enhancing welfare in both domestic and laboratory animals. The six studies presented highlight recent innovations in welfare assessment protocols, human-animal interaction dynamics, environmental enrichment, stress-reduction techniques, handling practices, and the incorporation of animal welfare considerations into global health policies. Collectively, these articles offer valuable insights into how scientific advancements and practical applications can address emerging welfare challenges.

In the article by researcher Dalmaou et al., a welfare assessment protocol specifically tailored to Japanese quail raised for meat is developed, providing a comprehensive method that incorporates indicators related to feeding, housing, health, and behavior. This study establishes the foundation for standardized welfare assessments in quail farming, identifying prevalent issues such as inadequate temperature control, suboptimal lighting, and overcrowding—factors with significant implications for welfare improvements across the industry.

Building on the theme of welfare assessment and enhancement, Mota-Rojas et al., delve into human-animal relationships (HAR) in *Bos indicus* cattle. By contrasting the reactive nature of *Bos indicus* with that of *Bos taurus* breeds, it underscores how positive HAR can reduce stress and improve welfare and productivity in cattle. This approach aligns with the quail welfare protocol, as both studies emphasize structured frameworks and the impact of human interaction on enhancing animal welfare.

Addressing the needs of laboratory animals, in this study Klein et al., investigate the impact of environmental enrichment on *Calomys callosus*, a rodent commonly used in research. Providing nesting materials and opportunities for exploration, this straightforward, low-cost enrichment strategy not only improved reproductive and health outcomes but also illustrated how welfare enhancements can lead to more reliable research findings. This enrichment approach parallels the emphasis on human-animal relationships (HAR) in enhancing welfare through environmental and social factors, underscoring the cumulative benefits for both animal welfare and scientific objectives.

Stress management techniques are examined in article presented by Sintuprom et al., which explore the use of clove oil as a stress-relieving additive for Siamese fighting fish during transport. By determining that a specific concentration (1 mg/L) of clove oil effectively reduces stress indicators, the study introduces a non-invasive, cost-effective method for managing transportation-induced stress. Its focus on measurable stress reduction aligns with the welfare protocols for quails and *Bos indicus*, reinforcing the importance of both physical and psychological care across species.

Al-Owaimer et al., investigate the impact of pre-slaughter handling practices on goats, emphasizing the significant role of gentle handling in reducing stress indicators and improving meat quality. Similar to the findings with *Bos indicus* cattle, this research underscores that handling intensity can directly influence animal welfare outcomes. Together, these studies advocate for handling practices that prioritize animal wellbeing, thereby enhancing both productivity and quality.

Finally, this Research Topic expands the discussion of animal welfare to a global policy level by examining the impact of the COVID-19 pandemic on the integration of animal welfare into health systems. This study by Huang et al., analyze the perspectives of civil society organizations regarding the incorporation of animal welfare into the WHO pandemic treaty, emphasizing the need for a coordinated, cross-sectoral approach that addresses animal welfare in conjunction with human health. This policy-oriented study reflects a forward-looking perspective, recognizing that integrating animal welfare into global health frameworks can help mitigate future public health challenges.

These studies, while distinct in their focus, converge on the theme of improving animal welfare through scientific research, humane handling practices, environmental enrichment, and integrative policy approaches. Collectively, they exemplify a holistic, interdisciplinary approach to animal welfare, offering practical solutions applicable across species and contexts to address the complex welfare needs of the future.
